# Supplementary material for: Clearance of persistent SARS-CoV-2 associates with increased neutralizing antibodies in advanced HIV disease post-ART initiation
Source: Nat Commun. 2024 Mar 15;15:2360. doi: 10.1038/s41467-024-46673-2 (PMC10943233; doi:10.1038/s41467-024-46673-2)
Supplement: Supplementary file 1 — Supplementary Information [file 41467_2024_46673_MOESM1_ESM.pdf]

Supplementary Material

Clearance of persistent SARS-CoV-2 associates with increased neutralizing antibodies in advanced HIV disease post-ART initiation

Karim et al.

Contents

Table S1: Participants enrolled in the study 2

Table S2: Participants for calculating infection period 2

Table S3: Advanced HIV disease participants vaccinated during study 2

Table S4: Control participants used to measure T cell responses 3

Table S5: Advanced HIV participants used to measure T cell responses 3

Table S6: Pfizer-vaccinated longitudinally tracked control participants 3

Table S7: Summary vaccinated participants (no advanced HIV disease) 4

Table S8: Participants from different infection periods 4

Figure S1: Antiretroviral drugs detected in plasma samples from the 5 participants with advanced HIV disease. 5

Figure S2: Substitutions or deletions in SARS-CoV-2 sequences of advanced HIV disease participants through time. 6

Figure S3: Effect of co-formulated TLD ART regimen on HIV and SARS-CoV-2 infection. 7

Figure S4: Neutralizing antibody response of participant plasma against autologous outgrown virus at different timepoints. 8

Figure S5: Kinetics of antibody isotype responses in plasma over time in advanced HIV disease participants. 9

Figure S6: Flow cytometry gating strategy and control participant responses. 10

Table S1: Participants enrolled in the study

	All 994	HIV- n=587 (59%)	HIV Suppressed* CD4** >200 n=259 (26%)	HIV viremic CD4** >200 n=35 (4%)	HIV viremic/suppressed CD4** <200 n=113 (11%)
Age (median, IQR)	41 (32-52)	42 (31-55)	42 (35-50)	34.5 (27-38)	41 (32-47)
Female	621 (62%)	347 (59%)	193 (75%)	22 (63%)	59 (52%)
SuppO2	143 (14%)	62 (11%)	36 (14%)	8 (23%)	37 (33%)
CD4 count (median, IQR)	774.5 (467-1035)	907.5 (676-1149)	695 (478-901.5)	433 (283-540)	65 (23-134)
Vaccination:					
Not vaccinated	493 (50%)	253 (43%)	127 (49%)	24 (69%)	89 (79%)
1 dose BNT162b2***	32 (3%)	18 (3%)	10 (4%)	1 (3%)	3 (3%)
1 dose Ad26.COV2.S	281 (28%)	210 (36%)	59 (23%)	6 (17%)	6 (5%)
2 doses BNT162b2	188 (19%)	106 (18%)	63 (24%)	4 (11%)	15 (13%)
Variant[#]:					
Ancestral	147 (15%)	86 (14%)	44 (17%)	8 (23%)	9 (8%)
Beta	135 (13%)	92 (16%)	24 (9%)	4 (11%)	15 (13%)
Delta	106 (11%)	52 (9%)	32 (12%)	3 (9%)	19 (16%)
Omicron BA.1, BA.2	97 (10%)	51 (9%)	23 (9%)	3 (9%)	20 (18%)
Omicron BA.5 related	53 (5%)	22 (4%)	14 (6%)	4 (11%)	13 (12%)
Omicron XBB	96 (10%)	65 (11%)	21 (8%)	2 (6%)	8 (7%)
No infection (controls)	360 (36%)	219 (37%)	101 (39%)	11 (31%)	29 (26%)
Comorbidities:					
Diabetes	108 (11%)	77 (13%)	26 (10%)	1 (3%)	4 (4%)
Hypertension	199 (20%)	129 (22%)	57 (22%)	4 (11%)	9 (8%)
Cardiovascular Disease	30 (3%)	25 (4%)	3 (1%)	0 (0%)	2 (2%)

*Median HIV viral load < 500. **Median CD4 count. ***At enrollment. [#]Variant determined by dominant circulating variant at enrollment.

Table S2: Participants for calculating infection period

	Advanced HIV disease	HIV-
Number of participants	24	24
Age (median, IQR)	37 (33-42)	37 (33-42)
Female	13 (54%)	13 (54%)
Viral Load (median, IQR)	40237 (5456-106518)	-
CD4	10 (5-21)	833 (614-988)

Table S3: Advanced HIV disease participants vaccinated during study

Participant	Sex	Age	Diagnosis	Infec. wave	Enrol. CD4	Enrol. HIV VL	1st Vacc. Date	Vacc. CD4	Vacc. HIV VL	Time to last positive
27	F	30-39	Sep 2020	D614G	6	34151	Sep 2021	92	40	215
96	M	40-49	Apr 2021	Beta	4	111883	Nov 2021	59	40	110
127	M	30-39	Mar 2021	Beta	12	8581	Sep 2021	120	40	207
209	F	30-39	Dec 2021	Omicron	24	423817	Apr 2022	31	661666	165
255	M	20-29	Sep 2021	Delta	2	12041	Jun-2022	42	17813	289

Table S4: Control participants used to measure T cell responses

Participant	Time-point (T)	Sex	Age Range	Sample date	Infection date*	Infection to sample (days)	Vaccine date	HIV status	CD4	CD8
17	1<br>2	F	60-69	Aug 20<br>Jul 21	Jul 20	33<br>369	Jun 21	Neg.	837<br>1025	472<br>693
21	1<br>2	F	30-39	Aug 20<br>Nov 21	Jul 20	24<br>479	Oct 21	PLWH	489<br>404	529<br>319
103	1<br>2	M	50-59	Jul 21<br>Mar 22	Jun 21	23<br>282	Feb 21	Neg.	904<br>1149	515<br>640
95	1<br>2	M	50-59	May 21<br>Nov 21	Jan 21	120<br>297	Oct 21	PLWH	576<br>419	990<br>853
109	1<br>2	M	40-49	Aug 21<br>Oct 21	Aug 21	20<br>83	Oct 21	PLWH	1181<br>1072	572<br>434

*By date of initial SARS-CoV-2 diagnosis.

Table S5: Advanced HIV participants used to measure T cell responses

Participant	Time-point (T)	Sex	Age Range	Sample date	Infection date*	Infection to sample (days)	Infection to clear. (days)	HIV VL	CD4	CD8
27	1<br>2	F	30-39	Apr 21<br>May 21	Sep 20	203<br>232	232	57<br>49	19<br>43	380<br>644
96	1<br>2	M	40-49	Jul 21<br>Aug 21	Apr 21	99<br>130	130	40<br>113	10<br>42	333<br>941
127	1<br>2	M	30-39	May 21<br>Oct 21	Mar 21	68<br>221	221	821<br>40	21<br>101	1180<br>1049
209	1<br>2	F	30-39	Apr 22<br>Jun 22	Dec 21	124<br>187	187	661666<br>550	31<br>252	170<br>797
255	1<br>2	M	20-29	Apr 22<br>Aug 22	Sep 21	238<br>364	293	148<br>40	54<br>16	691<br>203

*By date of initial SARS-CoV-2 diagnosis.

Table S6: Pfizer-vaccinated longitudinally tracked control participants

Participant	Sex	Age	Diagnosis	Infection wave	Enrol. CD4	Enrol. HIV VL	1st Vacc. Date	Vacc. CD4	Vacc. HIV VL	Time to last positive
9	F	40-49	Jun 2020	D614G	706	<40	Sep 2021	658	<40	25
21	F	30-39	Jul 2020	D614G	663	<40	Sep 2021	815	<40	9
69	F	40-49	Aug 2020	D614G	702	<40	Sep 2021	757	<40	11
123	F	20-29	Feb 2021	Beta	1551	-	Sep 2021	686	-	5
149	F	50-59	Jul 2021	Delta	211	-	Sep 2021	935	-	2

Table S7: Summary vaccinated participants (no advanced HIV disease)

	All (n=26)	HIV- (n=16)	PLWH (n=10)
Female	17 (65%)	11 (69%)	6 (60%)
Age (median, IQR)		42.5 (31-63)	47 (41-55)
Infecting Variant by Date			
Ancestral	16 (62%)	10 (63%)	6 (60%)
Beta	6 (23%)	4 (25%)	2 (20%)
Delta	4 (15%)	2 (13%)	2 (20%)
Vacc. CD4 (median, IQR)	830.5 (686-1003)	948 (765-1184.5)	707.5 (464-815)
Vacc. HIV VL (median, IQR)	-	-	40 (40-40)
Last dose to sample (median, IQR days)	24.5 (14-29)	27.5 (19-29.5)	15.5 (13-28)

.

Table S8: Participants from different infection periods

	Delta* n=10	BA.1 unvaccinated n=24	BA.1 vaccinated n=15	XBB unvaccinated n=8
Female	2 (20%)	16 (67%)	9 (60%)	5 (63%)
Age (median, IQR)	47 (41-56)	31.5 (26-49)	37 (32-60)	53 (37.5-67.5)
CD4 (median, IQR)	938 (671-1238)	772 (611-1055.5)	679 (584-904)	925 (723-1102)
HIV Positive	4 (40%)	8 (33%)	4 (27%)	2 (25%)
Diagnosis to sample (median, IQR days)	23 (22-26)	21.5 (17-26.5)	22 (17-23)	13 (9-17)
Last dose to sample (median, IQR days)	23 (21-31)	-	168 (148-220)	-

*Two participants vaccinated with 1 dose BNT162b2. Delta enrollments from 01 June 2021-31 Oct 2021. Omicron BA.1 enrollments from 01 Nov 2021-28 Feb 2022. Omicron BA.4/5 enrollments from 01 Mar 2022-31 Oct 2022. Omicron XBB enrollments from 01 Nov 2022-01 Jun 2023.

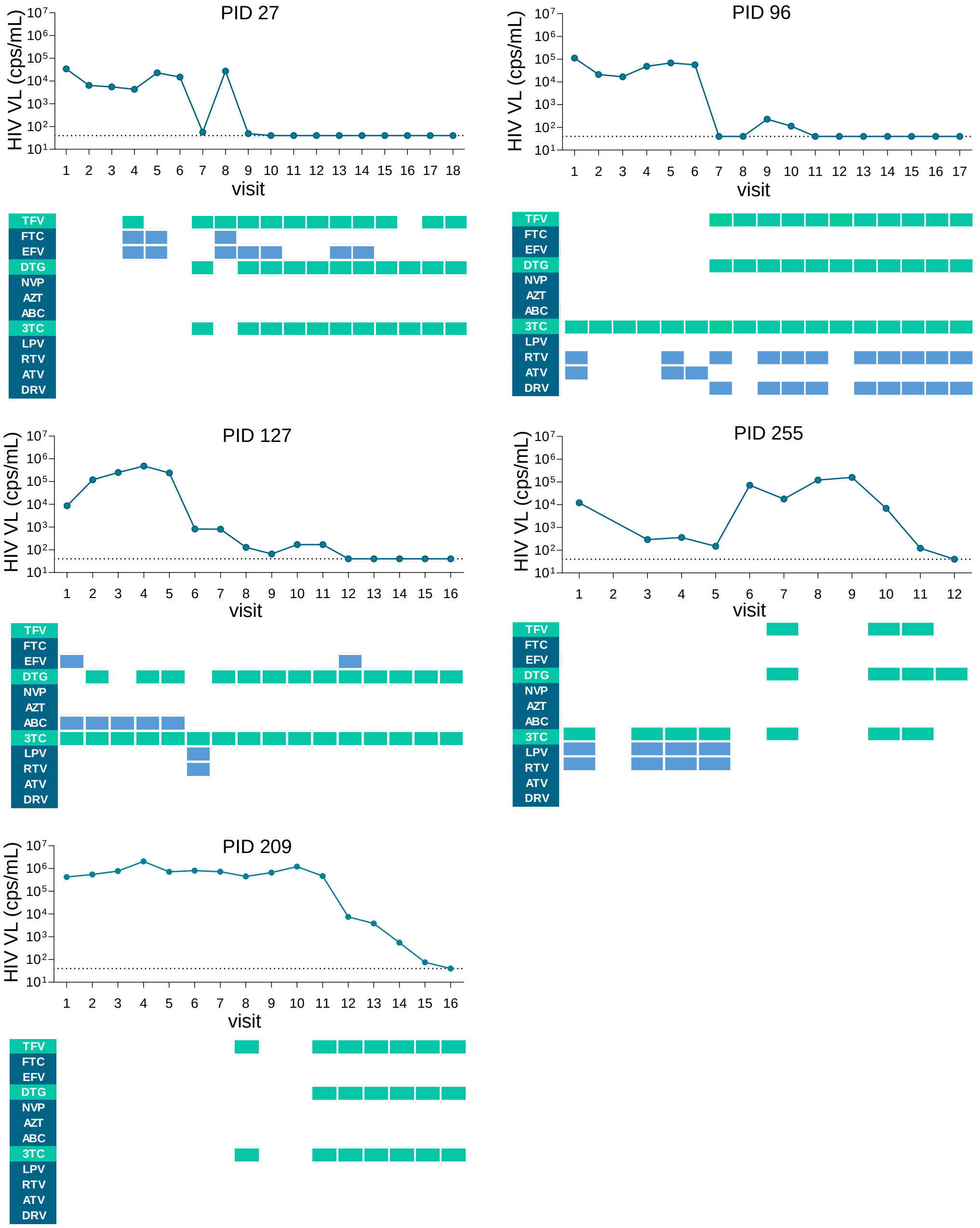

Figure S1: Antiretroviral drugs detected in plasma samples from the 5 participants with advanced HIV disease. ART components assayed by LC-MS/MS were the integrase inhibitor dolutegravir (DTG), the nucleotide reverse transcriptase inhibitor tenofovir (TFV), the nucleoside reverse transcriptase inhibitors emtricitabine (FTC), lamivudine (3TC), abacavir (ABC), and azidothymidine (AZT), the nonnucleoside reverse transcriptase inhibitors Efavirenz (EFV) and nevirapine (NVP), and the protease inhibitors lopinavir (LPV), ritonavir (RTV), atazanavir (ATV) and darunavir (DRV). X-axis is participant study visit and y-axis is HIV viral load as RNA copies/mL (above) or antiretroviral drug (below). Green or blue rectangles indicate the corresponding drug was detected at the timepoint at a level above 3 ng/mL, the limit of detection. Green rectangles indicate the presence of components of the regimen of TFV, 3TC, and DTG on which participants were initiated, and blue rectangles indicate components of other regimens.

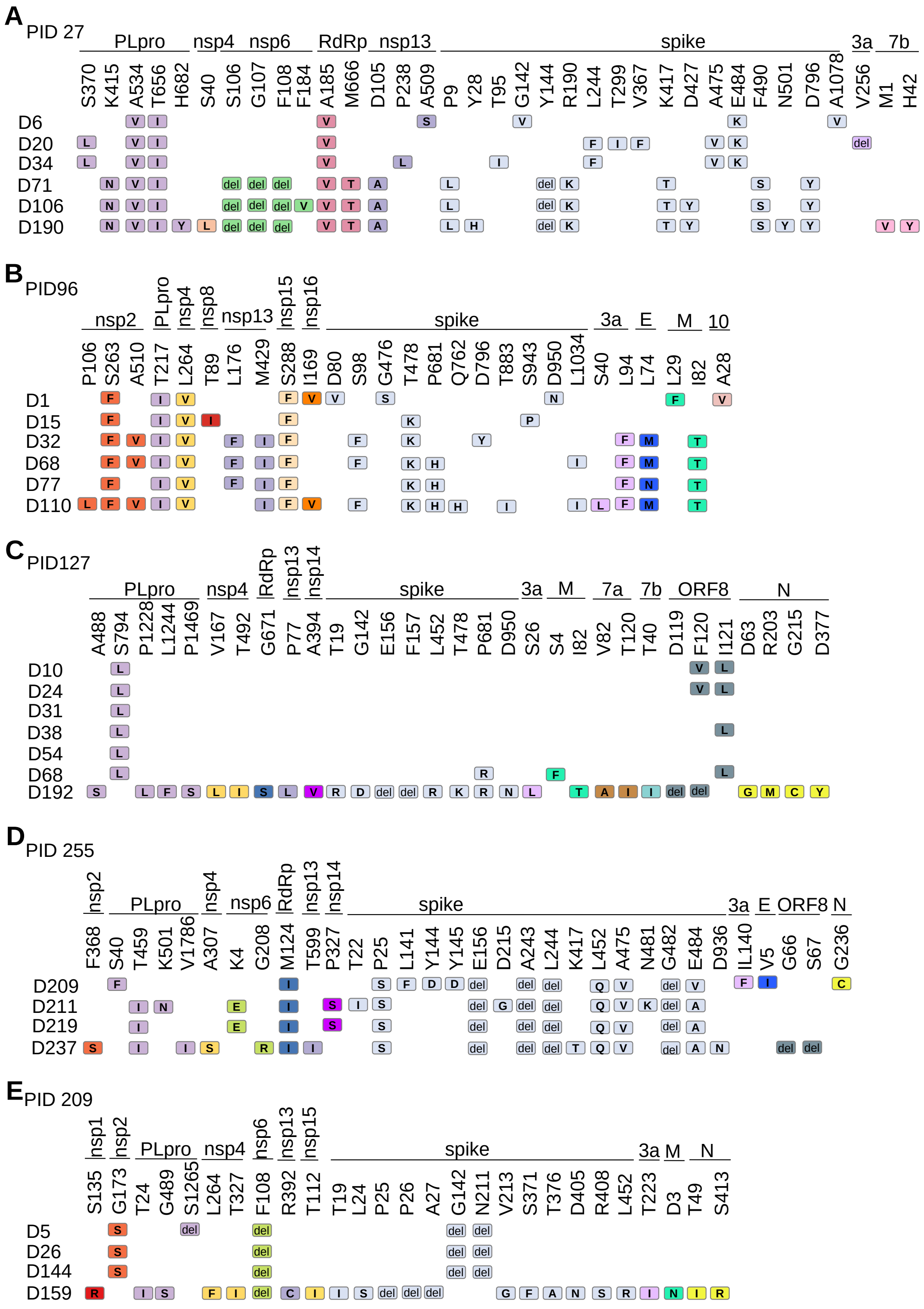

Figure S2: Substitutions or deletions in SARS-CoV-2 sequences of advanced HIV disease participants through time. Horizontal axis indicates the SARS-CoV-2 protein where substitution or deletion occurred relative to the infecting strain and vertical axis is the time post-diagnosis the viral isolate was obtained. Mutation calling in SARS-CoV-2 proteins performed using the Stanford Coronavirus Antiviral and Resistance Database (https://covdb.stanford.edu).

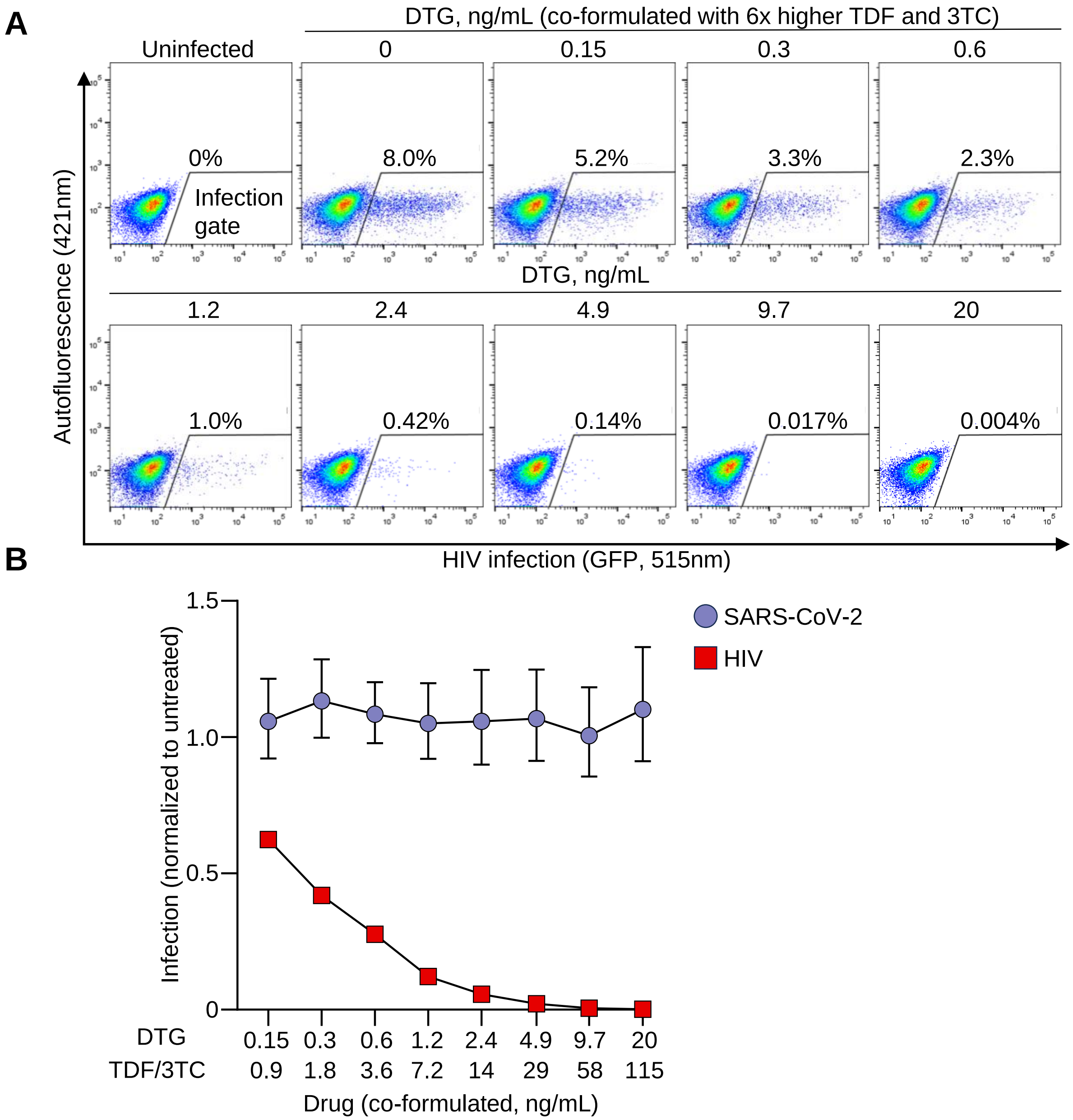

Figure S3: Effect of co-formulated TLD ART regimen on HIV and SARS-CoV-2 infection. A) RevCEM-GFP reporter cells were infected with NL4-3 HIV in the presence of co-formulated tenofovir disoproxil fumarate (TDF), lamivudine (3TC), dolutegravir (DTG), the TLD ART regimen. GFP positive infected cells were detected by flow cytometry in the indicated gate. DTG concentration indicated above each plot. TDF and 3TC levels were present at 6-fold higher concentrations relative to DTG. B) Fraction of HIV infected cells by flow cytometry and SARS-CoV-2 (ancestral D614G) infected foci by focus forming assay (see Materials and Methods) in the presence of the TLD regimen, normalized to infection in the absence of TLD. Shown are geometric means and geometric std from 6 (SARS-CoV-2) or 4 (HIV) replicates from two independent experiments.

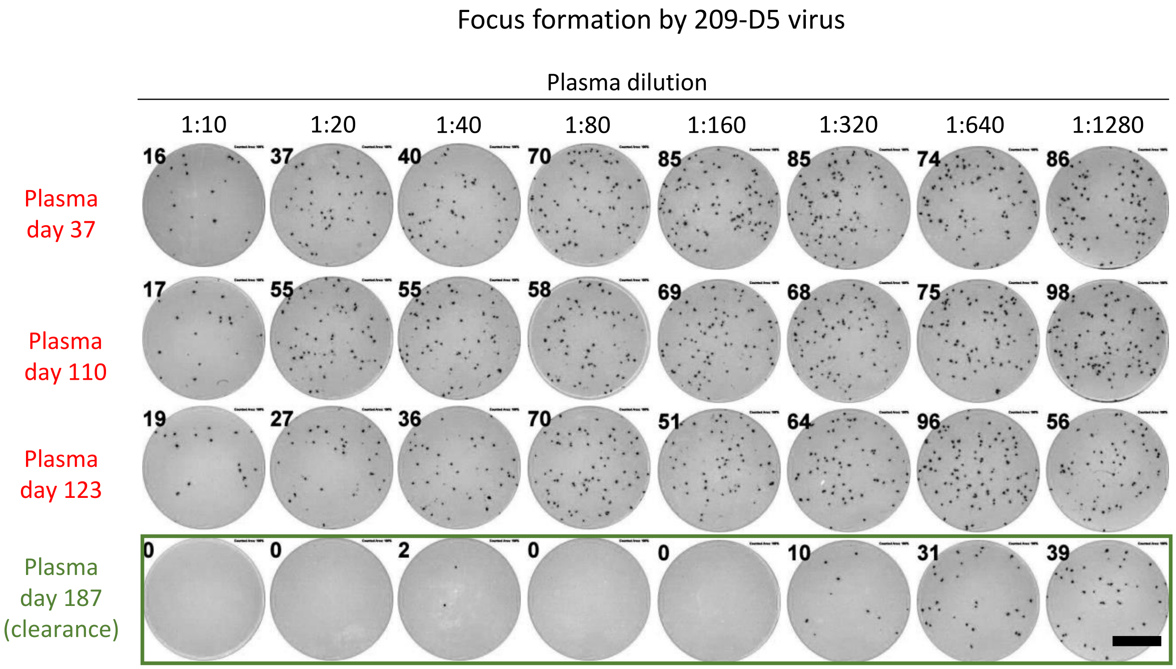

Figure S4: Neutralizing antibody response of participant plasma against autologous outgrown virus at different timepoints. Representative image showing infection foci in wells of a multi-well plate from a live virus focus forming assay of participant 209 plasma against autologous virus (209-D5). Plasma samples were from timepoints pre-SARS-CoV-2 clearance (D37, D110, D123) through to clearance (D187). Columns are plasma dilutions which range from 1:10 to 1:1280 and rows are plasma timepoint used. Bar is 2 mm.

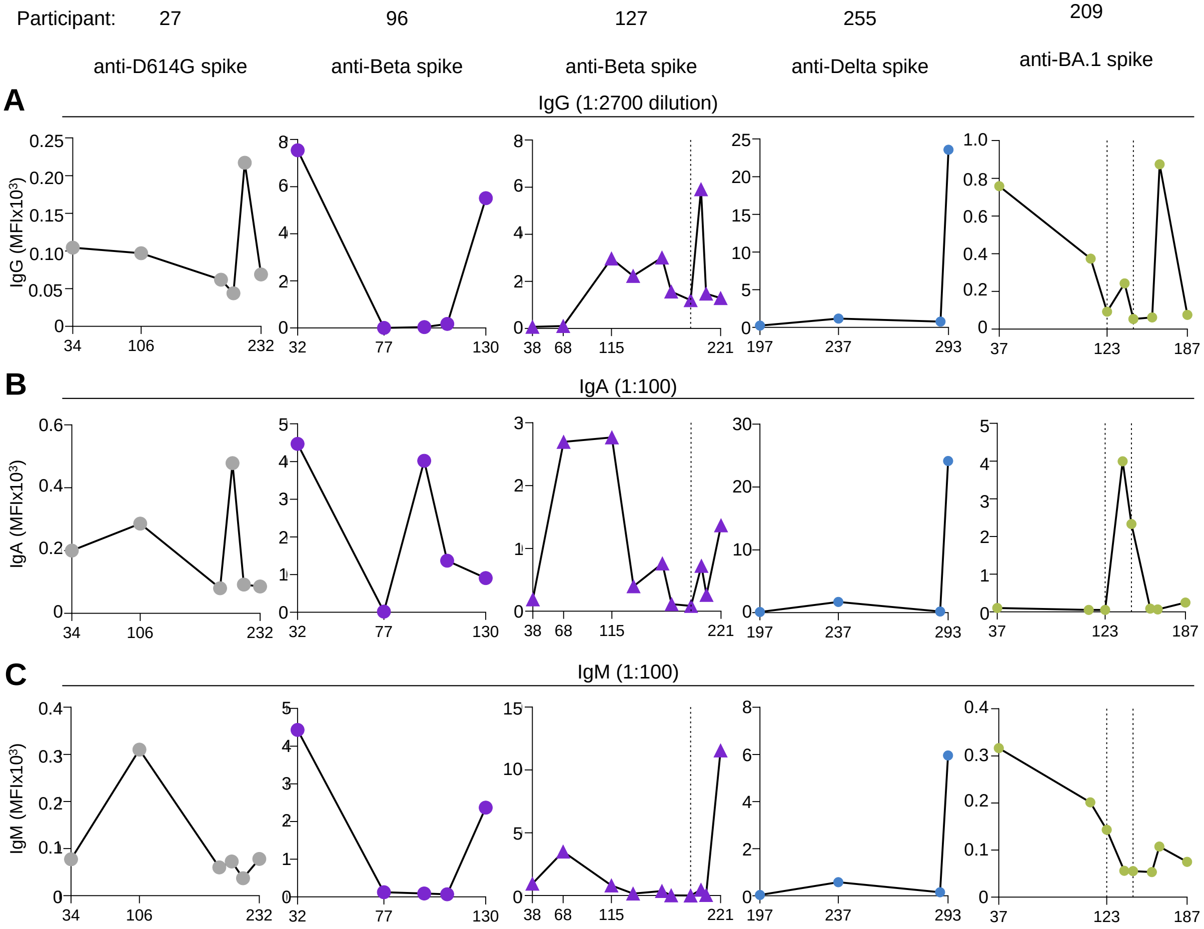

Figure S5: Kinetics of antibody isotype responses in plasma over time in advanced HIV disease participants. A) IgG responses to the spike matching the infecting variant, at a dilution of 1:2,700. B) IgM and C) IgA responses at a dilution of 1:100. Y-axis is mean fluorescence intensity (MFI) $\times 10^3$. X-axis is time in days post-SARS-CoV-2 diagnosis. One sample was analyzed per timepoint.

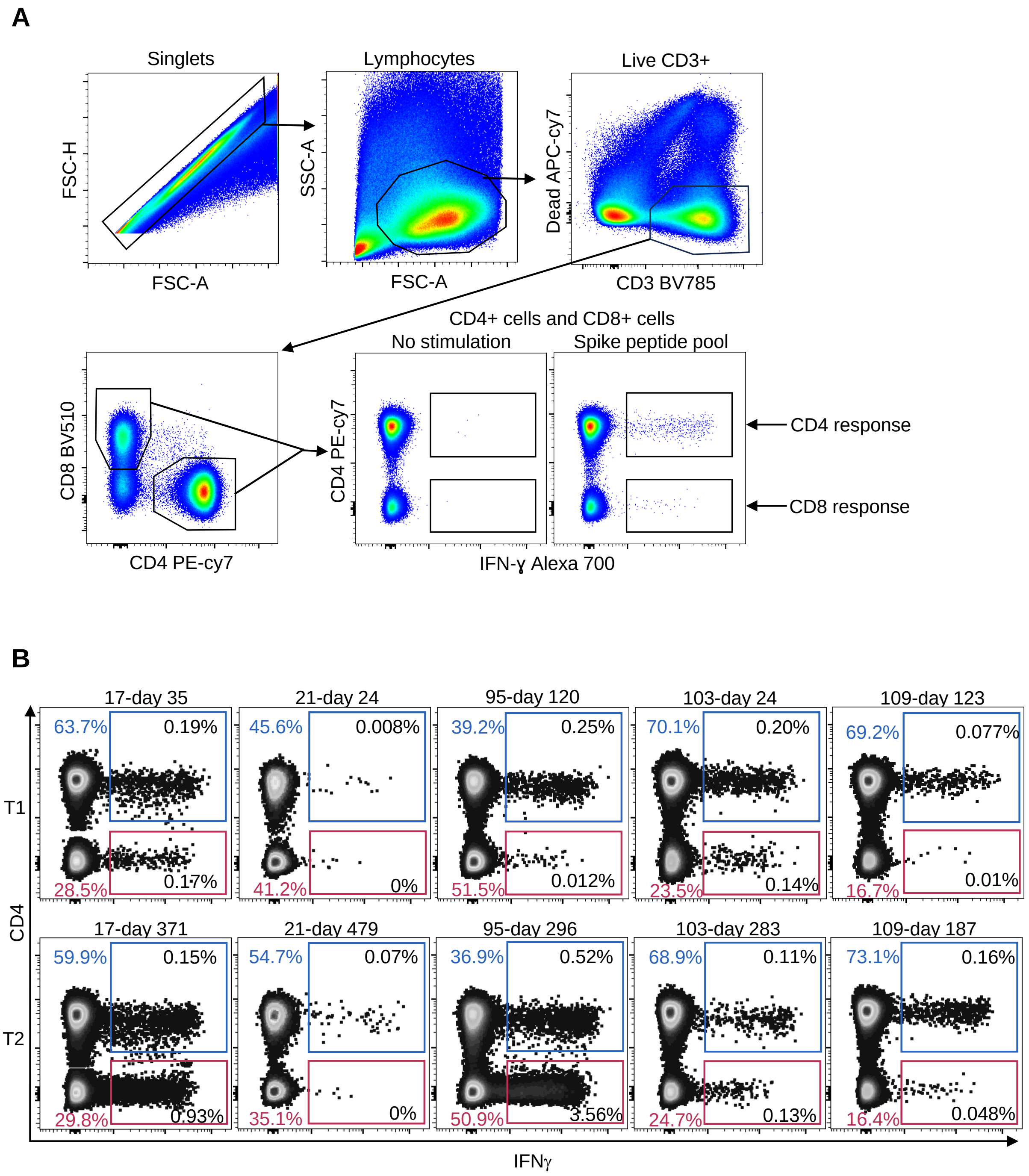

Figure S6: Flow cytometry gating strategy and control participant responses. A) Nested gating strategy to identify CD4+ and CD8+ T cell populations and IFN-γ production in response to Spike SARS-CoV-2 peptide pool in the CD4 and CD8 compartments. B) Expression of IFN-γ in CD4+ and CD8+ T cells upon stimulation with spike peptide pool in the five controls participants (17, 21, 95, 103 and 109) at two time points (top: Timepoint 1 was post-infection and pre-vaccination, and bottom: Timepoint 2 was post-vaccination). There was sufficient PBMC sample for one test per timepoint.
