# Supplementary material for: Clearance of persistent SARS-CoV-2 associates with increased neutralizing antibodies in advanced HIV disease post-ART initiation
Source: Nat Commun. 2024 Mar 15;15:2360. doi: 10.1038/s41467-024-46673-2 (PMC10943233; doi:10.1038/s41467-024-46673-2)
Supplement: Supplementary file 3 — Reporting Summary [file 41467_2024_46673_MOESM3_ESM.pdf]

nature portfolio

Corresponding author(s): Alex Sigal

Last updated by author(s): Feb 6, 2024

Reporting Summary

Nature Portfolio wishes to improve the reproducibility of the work that we publish. This form provides structure for consistency and transparency in reporting. For further information on Nature Portfolio policies, see our Editorial Policies and the Editorial Policy Checklist.

Statistics

For all statistical analyses, confirm that the following items are present in the figure legend, table legend, main text, or Methods section.

n/a	Confirmed	
☐	☒	The exact sample size (*n*) for each experimental group/condition, given as a discrete number and unit of measurement
☐	☒	A statement on whether measurements were taken from distinct samples or whether the same sample was measured repeatedly
☐	☒	The statistical test(s) used AND whether they are one- or two-sided<br>*Only common tests should be described solely by name; describe more complex techniques in the Methods section.*
☒	☐	A description of all covariates tested
☒	☐	A description of any assumptions or corrections, such as tests of normality and adjustment for multiple comparisons
☐	☒	A full description of the statistical parameters including central tendency (e.g. means) or other basic estimates (e.g. regression coefficient) AND variation (e.g. standard deviation) or associated estimates of uncertainty (e.g. confidence intervals)
☐	☒	For null hypothesis testing, the test statistic (e.g. *F*, *t*, *r*) with confidence intervals, effect sizes, degrees of freedom and *P* value noted<br>*Give P values as exact values whenever suitable.*
☒	☐	For Bayesian analysis, information on the choice of priors and Markov chain Monte Carlo settings
☒	☐	For hierarchical and complex designs, identification of the appropriate level for tests and full reporting of outcomes
☒	☐	Estimates of effect sizes (e.g. Cohen's *d*, Pearson's *r*), indicating how they were calculated

Our web collection on statistics for biologists contains articles on many of the points above.

Software and code

Policy information about availability of computer code

Data collection	Participant data was collected using REDCap version 11.1.29
Data analysis	All statistics and fitting were performed using custom code in MATLAB v.2019b. Sequences were analysed using the Standford Corona Virus Antiviral and Resistance Database (https://covdb.stanford.edu/sierra/sars2/by-sequences/) with HTML as output. Figures were prepared using GraphPad Prism version 10.

For manuscripts utilizing custom algorithms or software that are central to the research but not yet described in published literature, software must be made available to editors and reviewers. We strongly encourage code deposition in a community repository (e.g. GitHub). See the Nature Portfolio guidelines for submitting code & software for further information.

Data

Policy information about availability of data

All manuscripts must include a data availability statement. This statement should provide the following information, where applicable:

- Accession codes, unique identifiers, or web links for publicly available datasets
- A description of any restrictions on data availability
- For clinical datasets or third party data, please ensure that the statement adheres to our policy

Sequences of isolated SARS-CoV-2 used in this study have been deposited in GISAID and GenBank with accession numbers as follows: D614G (B.1 lineage),

EPI_ISL_602626.1 (GISAID), OP090658 [https://www.ncbi.nlm.nih.gov/nuccore/OP090658] (GenBank). BA.1 (B.1.1.529.1), EPI_ISL_7886688, OP090659, [https://www.ncbi.nlm.nih.gov/nuccore/OP090659]. BA.4, EPI_ISL_12268495.2, OP093374, [https://www.ncbi.nlm.nih.gov/nuccore/OP093374]. BA.5, EPI_ISL_12268493.2, OP093373, [https://www.ncbi.nlm.nih.gov/nuccore/OP093373]. XBB.1.5, EPI_ISL_17506815, OR782922, [https://www.ncbi.nlm.nih.gov/nuccore/OR782922]. BA.2.86, EPI_ISL_18226980, OR775659, [https://www.ncbi.nlm.nih.gov/nuccore/OR775659]. Beta (B.1.351), EPI_ISL_678615, OR936719, [https://www.ncbi.nlm.nih.gov/nuccore/OR936719]. Delta (B.1.617.2), EPI_ISL_3118687, OR936720, [https://www.ncbi.nlm.nih.gov/nuccore/OR936720]. 0027-D6, EPI_ISL_15541746, OR936722, [https://www.ncbi.nlm.nih.gov/nuccore/OR936722]. 0027-D20, EPI_ISL_15541747, OR936723, [https://www.ncbi.nlm.nih.gov/nuccore/OR936723]. 0027-D34, EPI_ISL_15541748, OR936751, [https://www.ncbi.nlm.nih.gov/nuccore/OR936751]. 0027-D71, EPI_ISL_15541749, OR936770, [https://www.ncbi.nlm.nih.gov/nuccore/OR936770]. 0027-D106, EPI_ISL_15541750, OR936771, [https://www.ncbi.nlm.nih.gov/nuccore/OR936771]. 0027-D190, EPI_ISL_2397313, OR936772, [https://www.ncbi.nlm.nih.gov/nuccore/OR936772]. 0096-D1, EPI_ISL_14666761, OR936774, [https://www.ncbi.nlm.nih.gov/nuccore/OR936774]. 0096-D15, EPI_ISL_14666763. 0096-D32, EPI_ISL_13986492, OR936848, [https://www.ncbi.nlm.nih.gov/nuccore/OR936848]. 0096-D68, EPI_ISL_18030390, OR939248, [https://www.ncbi.nlm.nih.gov/nuccore/OR939248]. 0096-D77, EPI_ISL_18030391, OR939249, [https://www.ncbi.nlm.nih.gov/nuccore/OR939249]. 0096-D110, EPI_ISL_14666766. 0127-D10, EPI_ISL_16508746, OR939365, [https://www.ncbi.nlm.nih.gov/nuccore/OR939365]. 0127-D24, EPI_ISL_16508747, OR939364, [https://www.ncbi.nlm.nih.gov/nuccore/OR939364]. 0127-D31, EPI_ISL_16508748, OR939446, [https://www.ncbi.nlm.nih.gov/nuccore/OR939446]. 0127-D38, EPI_ISL_16508749, OR939591, [https://www.ncbi.nlm.nih.gov/nuccore/OR939591]. 0127-D54, EPI_ISL_18030392, OR939646, [https://www.ncbi.nlm.nih.gov/nuccore/OR939646]. 0127-D68, EPI_ISL_16508751, OR939648, [https://www.ncbi.nlm.nih.gov/nuccore/OR939648]. 0127-D192, EPI_ISL_14666773, OR939692, [https://www.ncbi.nlm.nih.gov/nuccore/OR939692]. 0255-D209, EPI_ISL_14599778, OR939724, [https://www.ncbi.nlm.nih.gov/nuccore/OR939724]. 0255-D211, EPI_ISL_14599779, OR939726, [https://www.ncbi.nlm.nih.gov/nuccore/OR939726]. 0255-D219, EPI_ISL_14599780, OR939727, [https://www.ncbi.nlm.nih.gov/nuccore/OR939727]. 0255-D237, EPI_ISL_13986497, OR939737, [https://www.ncbi.nlm.nih.gov/nuccore/OR939737]. 0209-D5, EPI_ISL_18030393, OR939740, [https://www.ncbi.nlm.nih.gov/nuccore/OR939740]. 0209-D26, EPI_ISL_18030394, OR939739, [https://www.ncbi.nlm.nih.gov/nuccore/OR939739]. 0209-D144, EPI_ISL_12970433, OR939741, [https://www.ncbi.nlm.nih.gov/nuccore/OR939741]. 0209-D159, EPI_ISL_14666777, OR939743, [https://www.ncbi.nlm.nih.gov/nuccore/OR939743].

Research involving human participants, their data, or biological material

Policy information about studies with human participants or human data. See also policy information about sex, gender (identity/presentation), and sexual orientation and race, ethnicity and racism.

Reporting on sex and gender	Sex and gender was not considered in the study design, how this is information is collected and is based on self report by participants. Due to the nature of the pandemic, we did not select for sex/gender and used participant samples that were available at the time of experiments that were within the inclusion criteria.
Reporting on race, ethnicity, or other socially relevant groupings	Participants enrolled in the study were based on the patient population presenting to hospitals and clinics in Durban, South Africa.
Population characteristics	Participant characteristics are listed in Table S1-S8
Recruitment	SARS-CoV-2 infected participants, blood samples were obtained from adults with PCR-confirmed SARS-CoV-2 infection who were enrolled in a prospective cohort study approved by the Biomedical Research Ethics Committee at the University of KwaZulu-Natal.
Ethics oversight	The Biomedical Research Ethics Committee at the University of KwaZulu-Natal approved the prospective cohort study. (reference BREC/00001275/2020). Investigators were blinded to participant information. The Omicron/BA.1 virus was isolated from residual swab used for diagnostic testing (Witwatersrand Human Research Ethics Committee). The nasopharyngeal swab for isolation of the Omicron XBB.1.5 subvariant was collected after written informed consent as part of the COVID-19 transmission and natural history in KwaZulu-Natal, South Africa: Epidemiological Investigation to Guide Prevention and Clinical Care in the Centre for the AIDS Programme of Research in South Africa (CAPRISA) study and approved by the Biomedical Research Ethics Committee at the University of KwaZulu–Natal (reference BREC/00001195/2020, BREC/00003106/2021).

Note that full information on the approval of the study protocol must also be provided in the manuscript.

Field-specific reporting

Please select the one below that is the best fit for your research. If you are not sure, read the appropriate sections before making your selection.

[x] Life sciences [ ] Behavioural & social sciences [ ] Ecological, evolutionary & environmental sciences

For a reference copy of the document with all sections, see nature.com/documents/nr-reporting-summary-flat.pdf

Life sciences study design

All studies must disclose on these points even when the disclosure is negative.

Sample size	Samples size was not predetermined. We used samples available that met the inclusion/exclusion criteria
Data exclusions	We did not exclude participants that met inclusion criteria
Replication	Repeated in independent experiments on different days in sets of paired experiments. For self neutralization, repeats were performed on the same day, in the same experiment. Geometric mean of replicate samples was used. All repeat experiments were successful
Randomization	Participants were allocated into groups based on their reported on HIV and vaccination status

Blinding	The study is an observational cohort study, investigators were blinded to participant data during data collection. De-identified data was made available to investigators.

Reporting for specific materials, systems and methods

We require information from authors about some types of materials, experimental systems and methods used in many studies. Here, indicate whether each material, system or method listed is relevant to your study. If you are not sure if a list item applies to your research, read the appropriate section before selecting a response.

Materials & experimental systems

n/a	Involved in the study
☐	☒ Antibodies
☐	☒ Eukaryotic cell lines
☒	☐ Palaeontology and archaeology
☐	☒ Animals and other organisms
☒	☐ Clinical data
☒	☐ Dual use research of concern
☒	☐ Plants

Methods

n/a	Involved in the study
☒	☐ ChIP-seq
☐	☒ Flow cytometry
☒	☐ MRI-based neuroimaging

Antibodies

Antibodies used	For virus neutralization assays, foci were stained with a rabbit anti-spike monoclonal antibody (BS-R2B12, GenScript A02058) at 0.5 µg/mL and a secondary goat anti-rabbit HRP conjugated antibody (Abcam ab205718) was added at 1 µg/mL . For the T-cell assays, CD28 (clone 28.2) and CD49 (clone L25) (1 µg/mL each; BD Biosciences) was used. Cells were stained with CD3 BV785 (OKT3, Biolegend), CD4 PE-Cy7 (L200, BD Biosciences), CD8 BV510 (RPA-8, Biolegend) and IFN-g Alexa 700 (B27, BD Biosciences).<br>For the Luminex based isotyping of antibody responses, 50 µL of 0.65 µg phycoerythrin (PE) conjugated secondary detection antibodies were added (Mouse-Anti Human IgM-PE (Southern Bioteck, cat no 9020-09), Goat Anti Human IgA-RPE (Bio-rad, Ref 205009) and total IgG (Goat Anti-Human IgG-fc (Invitrogen, 12-4998-82) was used.
Validation	BS-R2B12, GenScript A02058:<br>https://www.genscript.com/antibody/A02058-MonoRab_SARS_CoV_2_Spike_S1_Antibody_BS_R2B12_mAb_Rabbit.html<br>Abcam ab205718:<br>https://www.abcam.com/products/secondary-antibodies/goat-rabbit-igg-hl-hrp-ab205718.html

Eukaryotic cell lines

Policy information about cell lines and Sex and Gender in Research

Cell line source(s)	The H1299-E3 (H1299-ACE2, clone E3) cell line used in the live virus infections he H1299-E3 (H1299-ACE2, clone E3) cell line was derived from H1299 as described in our previous work. H1299 was a gift from M. Oren, originally obtained from ATCC, (CRL-5803). HEK293 cells (CRL-1572) used to produce HIV were from ATCC. The RevCEM-GFP HIV infection reporter cell line was obtained from the AIDS Research and Reference Reagent Program, National Institute of Allergy and Infectious Diseases, National Institutes of Health from Y. Wu and J. Marsh. The cell line was subcloned (see description in ref.91) to increase the maximum fraction of cells with GFP fluorescence upon HIV infection and clone B8 used for the assays.
Authentication	Cell lines have not been authenticated.
Mycoplasma contamination	The cell lines have been tested for mycoplasma contamination and are mycoplasma negative.
Commonly misidentified lines (See ICLAC register)	None

Animals and other research organisms

Policy information about studies involving animals; ARRIVE guidelines recommended for reporting animal research, and Sex and Gender in Research

Laboratory animals	Golden Syrian hamster (Mesocricetus auratus), 4-5 weeks old were used in this study
Wild animals	No wild animals were used in this study
Reporting on sex	Sex was not considered in the study design and we used animals available for the experiment. n=30 hamsters (19 female, 11 male, 15 animals per experiment with 2 experiments performed) were used. 6 animals were used per infection condition

Field-collected samples	No field collected samples were used in this study
Ethics oversight	Experimental work was approved by the Animal Ethics Committee at the University of KwaZulu Natal (reference: REC/00004197/2022).

Note that full information on the approval of the study protocol must also be provided in the manuscript.

Plants

Seed stocks	N/A
Novel plant genotypes	N/A
Authentication	N/A

Flow Cytometry

Plots

Confirm that:

- [x] The axis labels state the marker and fluorochrome used (e.g. CD4-FITC).
- [x] The axis scales are clearly visible. Include numbers along axes only for bottom left plot of group (a 'group' is an analysis of identical markers).
- [x] All plots are contour plots with outliers or pseudocolor plots.
- [x] A numerical value for number of cells or percentage (with statistics) is provided.

Methodology

Sample preparation	Cryopreserved peripheral blood mononuclear cells (PBMC) were thawed, washed, and rested for 4 hours in RPMI 1640 (Sigma-Aldrich) supplemented with 10% heat inactivated FBS. After resting, cells were seeded in a 96-well V-bottom plate at ~0.5 to 1 x106 cells/well. Cells were stimulated with custom made SARS-CoV-2 mega pools spanning the entire Spike protein of the ancestral, Beta, Delta or Omicron variants (1 μg/mL), provided by Dr Alessandro Sette (La Jolla Institute for Immunology, USA). All stimulations were performed in the presence of Brefeldin A (10 μg/mL, Sigma-Aldrich) and co-stimulatory antibodies against CD28 (clone 28.2) and CD49 (clone L25) (1 μg/mL each; BD Biosciences). As a negative control, PBMC were incubated with co-stimulatory antibodies, Brefeldin A and an equimolar amount of DMSO. After 16 hours of stimulation, cells were washed, stained with LIVE/DEAD™ Fixable Near-IR Stain (Invitrogen) and subsequently fixed and permeabilized using Cytofix/Cytoperm buffer (BD Biosciences). Cells were then stained with CD3 BV785 (OKT3, Biolegend), CD4 PE-Cy7 (L200, BD Biosciences), CD8 BV510 (RPA-8, Biolegend) and IFN- Alexa 700 (B27, BD Biosciences). After staining, cells were washed and fixed in 1% Paraformaldehyde (ThermoFisher Scientific).
Instrument	Samples were acquired on a BD Fortessa flow cytometer.
Software	Acquisition: FACSDiva software (BD). Analysis: FlowJo (v10, FlowJo LLC).
Cell population abundance	Cell population abundance varied between participants in different immune suppression states. They are described per participant in Figures 3 and S6B.
Gating strategy	Gating strategy presented in Figure S6A and involved gating for singlets, then lymphocytes, then CD3+ cells, then CD4 and CD8 T cell subsets which were combined (OR gate).

- [x] Tick this box to confirm that a figure exemplifying the gating strategy is provided in the Supplementary Information.
